# Characterization of Prickly Pear Peel Flour as a Bioactive and Functional Ingredient in Bread Preparation

**DOI:** 10.3390/foods9091189

**Published:** 2020-08-27

**Authors:** Lucia Parafati, Cristina Restuccia, Rosa Palmeri, Biagio Fallico, Elena Arena

**Affiliations:** Di3A, Dipartimento di Agricoltura, Alimentazione e Ambiente, University of Catania, via S. Sofia 100, 95123 Catania, Italy; lucia.parafati@unict.it (L.P.); crestu@unict.it (C.R.); bfallico@unict.it (B.F.); earena@unict.it (E.A.)

**Keywords:** food byproducts, *Opuntia ficus indica*, functional ingredient, phenolic compounds, antioxidant activity, bread

## Abstract

The aim of the present research was to evaluate the addition of prickly pear peel flour (PPPF) to bread dough as a source of nutrient and bioactive compounds. The PPPF’s physical, chemical and nutritional composition was evaluated, as well as its content of bioactive compounds betalains, and flavonoids. The characterization evidenced high fiber and carbohydrate contents and an elevated amount of polyphenols and betalain compounds. The PPPF was then added at different concentrations (5, 10, 15, 20, 50%, *w/w*) to bread formulations as a potential functional ingredient. All concentrations, except 50% PPPF, evidenced good leavening dough properties and were then tested for baking. In relation to the content of added PPPF, the amount of betalains, representing bioactive compounds, remained high even after the baking process, suggesting a protective matrix effect. Among the different formulations, those containing PPPF at 10% showed the highest values in terms of the leavening dough capacity and bread specific volume and received the best sensory evaluation score.

## 1. Introduction

Bread plays a very important role in the Mediterranean food tradition, being a primary component in the diet for a long time. Nowadays consumers are more attentive to the consumption of “healthy foods”. For this reason, the bakery sector has started to develop a wide range of baked products enriched with bioactive ingredients, such us dietary fibers, antioxidants and phenolic compounds [1,2]. Research activity also focused on the glycemic properties of various commercially available breads and found that breads rich in natural fibers (e.g., beta glucans) and proteins improve glycemic control and insulin sensitivity [3].

The prickly pear fruits (*Opuntia ficus indica*) are widespread in Italy, especially in Sicily where they are consumed as fresh fruit or widely used for the preparation of jam and traditional sweets. The fruit pulp possesses valuable nutritional properties, due to the high level of minerals, vitamins, and antioxidants. The consumption of cactus pear fruit positively affects the body’s redox balance, decreases oxidative damage to lipids, and improves antioxidant status in healthy humans [4,5]. Moreover, the fruit infusion also shows antidiuretic effects [6].

The fruit peel is generally discarded, used as soil improver or exploited by the livestock feed industry. Since the peel constitutes about 48% of the total fruit weight, it represents a concern for the food processing industry for the large amount of waste and the economic costs for its disposal. The proper utilization of this by-product could reduce waste disposal problems and serve as a potential new source of bioactive compounds.

The transformation of fruit by-products in flour to be used in functional foods has already been hypothesized by different authors ([7,8] and the references therein). An experimental study carried out on rats evidenced that the addition of cactus flour to a western-style diet was effective to attenuate the risk parameters for the occurrence of metabolic syndromes such as sub fraction high cholesterol levels and glucose tolerance [9]. Due to the high nutrition value and the important quantity of fiber [10], antioxidants [5] and flavonoids [11], Opuntia peel has been considered a functional ingredient for improving the physicochemical, structural and nutritional properties of cooked sausages [12] and gluten-free snacks [13]. The study of [14] investigated the utilization of very small quantities of prickly pear peels (0.5, 1 and 2%) for the improvement of pan bread quality and reported that this vegetable matrix can increase the shelf-life of pan bread and decrease staling.

However, by-products obtained by fruits and vegetables possess antimicrobial properties due to their antioxidant activity and to the high content of total polyphenols and betalains [15,16,17], therefore, the addition of such ingredients in leavened bakery products, such as bread, could negatively affect the ability of *Saccharomyces cerevisiae* baker’s yeast to leave the dough.

Therefore, the aims of this work were: to characterize prickly pear peel flour’s (PPPF) physical, chemical, nutritional and antioxidant properties; to evaluate the effects of PPPF addition, at different concentrations in the dough formulation, on the physical and chemical properties as well as on technological parameters of dough and bread; to create a sensory profile of bread added with PPPF, in comparison to traditional bread; to measure the amount of bioactive compounds remaining after bread cooking.

## 2. Materials and Methods

### 2.1. Preparations of Prickly Pear Peel Flour

Thornless prickly pear fruits (*Opuntia ficus indica*) of first flowering yellow cultivar ‘‘Agostani’’, were bought from a commercial orchard in Catania (Sicily) and immediately transported in the laboratory into a cardboard box. The fruits, selected for similar ripening stages, were superficially washed with sterile distilled water (SDW), and left to dry at room temperature (25 °C).

In total, 10 kg of fruits were hand-peeled by using a knife and the peel obtained (4 kg/fresh weight) were dried at 42 ± 1 °C for 48 h. After drying treatment, the peel was grinded and the obtained flour was ground down using 38 mesh sieves (0.5 mm) to standardize the particle size. The obtained prickly pear peel flour’s (PPPF) moisture content and activity water (Aw) were immediately analyzed and then vacuum packed and stored at −18 °C for the subsequent analysis.

### 2.2. Physicochemical and Nutritional Characterization of Prickly Pear Peel Flours (PPPF)

The moisture content of PPPF, expressed as moisture percentage (M%), was determined by drying at 105 °C until a constant weight was achieved with an electronic moisture balance (Eurotherm, Gibertini^®^, Novate Milanese, Italy). The Aw was measured, according to the manufacturer’s instructions, using the hygrometric method at 20 °C by Aqualab Vapor Sorption (Decago Device, Pullman, WA, USA). The flour’s protein, ash, lipid and total dietary fiber content was also analyzed according to the standard methods described by the Association of Official Analytical Chemist (AOAC, 2007) [18].

The total carbohydrate content was obtained by differences and calculated by the following formula:

Total carbohydrate % = 100 − (protein % + lipid % + moisture % + ash %)

### 2.3. Evaluation of Prickly Pear Peel Flour (PPPF) Bioactive Compounds

An aqueous extract of PPPF was obtained by dissolving the PPPF in hot water (90 °C), as described by [19], and its total polyphenol and antioxidant activity was analyzed. In brief, 100 mL of hot water was used as aqueous solvent for the treatment of 50 g of PPPF, prepared as previously described.

### 2.4. Total Polyphenol Content

The total polyphenol content (TPC) was evaluated using the Folin–Ciocalteau method as reported by Vazquez-Roncero et al. [20], with some modifications. An amount of 250 µL of extract was mixed with 1.25 mL of Folin–Ciocalteau reagent (FC) and incubated for 3 min, then 2.5 mL of 20% sodium carbonate (Na_2_CO_3_) was added. The mixture was brought to a final volume of 25 mL and let to react in the dark for 1 h. After the incubation, the absorbance was measured at 725 nm, using a Perkin Elmer lambda 25 Ultraviolet-Visible spectrometer (PerkinElmer Inc, Waltham, WA, USA).

The results in terms of the total polyphenols content were expressed as mg/g of PPPF (dry matter) of gallic acid equivalents (GAE), and the standard curve was obtained with eleven gallic acid concentrations (range from 0 to 80 mg/mL).

### 2.5. Total Flavonoids

The aluminum chloride colorimetric method reported by Lin and Tang [21] was used, with slight modifications, to evaluate the total flavonoids content of the sample. In brief, 1 g of PPPF was dissolved in 10 mL of deionized water and let to stir at room temperature for 30 min. An amount of 0.5 mL of the obtained extract, filtered using a 0.45 µm pore-size membrane filter, was mixed with 1.5 mL of 95% alcohol, 0.1 mL of 10% aluminum chloride hexahydrate (AlCl_3_·6H_2_O), 0.1 mL of 1 M potassium acetate (CH_3_COOK), and 2.8 mL of deionized water. The control sample (blank) was prepared by substituting the sample with deionized water. Each reaction mixture was incubated at room temperature for 40 min, and the absorbance was measured spectrophotometrically at 415 nm against the blank. Quercetin 3-β-d-glucoside was used as a standard to create a seven-point standard curve (0–50 mg/L) and the results were expressed as mg/100 g of PPPF (dry matter) of quercetin equivalents (QE).

### 2.6. Betalains

Betacyanins and betaxanthins were evaluated following the method reported by Ruiz-Gutierrez et al. [22], with slight modifications. Briefly, 10 g of sample was diluted in 100 mL of deionized water and homogenized with Ultra Turrax T18 equipment (IKA ULTRA-TURRAX^®^, Wilmington, NC, USA). After homogenization, the sample was centrifuged at 10,000× *g* at 4 °C for 10 min in a centrifuge (ALC 4239R) and the obtained supernatant was filtered by a 0.45 μm pore-size membrane filter (Millipore^®^, Burlington, MA, USA). The extract was analyzed spectrophotometrically at 536 nm and 481 nm, for betacyanin and betaxanthin, respectively, using the molecular weight (Mw) and molar extinction coefficient (ε) in water of betanin (Mw = 550 g/mol; ε = 60,000 L/mol) and indicaxanthin (Mw = 308 g/mol; ε = 48,000 L/mol). All the measurements were conducted in triplicate and the results were expressed as the mg of betacyanin and betaxanthin in 100 g of PPPF (dry matter).

### 2.7. Evaluation of Antioxidant Activity

The antioxidant activity was evaluated on the PPPF extract using the 2,2-diphenyl-1-picrylhydrazyl (DPPH) radical scavenging activity method reported by Brand-Williams et al. [23], with some modifications. The assay was conducted by mixing 3 mL of methanol DPPH (2,2-diphenyl-1-picrylhydrazyl) solution (100 µM) with 50 µL of the aqueous extract, prepared as described before, homogenized and incubated in the dark for one hour at 25 °C. The control sample (blank) was prepared in the same way but the same amount of extract was replaced with methanol. At the end of the reaction period the absorbance of each sample was read at 515 nm using a Perkin Elmer lambda 25 Uv-Vis spectrometer. Trolox was used as a standard to create an eight-point standard curve (0–75 mg/L) and the antioxidant capacity was expressed as the mg/kg of the PPPF (dry sample) of trolox equivalents.

### 2.8. Evaluation of Doughs Containing Prickly Pear Peel Flour (PPPF)

In order to evaluate the influences of PPPF on dough performances, different composite flours were prepared by mixing commercial durum wheat flour (moisture 15%, ash 0.90%, protein 11.50%, hydratation 50%, W 190) with 5, 10, 15, 20 and 50% PPPF. The control was made using only durum wheat flour. The obtained composite flours were evaluated on their water and oil binding capacities described below and then used for the preparation of different doughs.

### 2.9. Water and Oil Binding Capacities of the Prickly Pear Peel Flour (PPPF) of Composite Flours

The water binding capacity (WBC) or oil binding capacity (OBC) of composite flours, prepared as described before, were evaluated as reported by Kahraman et al. [24] with minor modifications. In detail, 2 g of each sample was mixed with 24 mL of SDW and let to stir for 60 min at room temperature. The samples were then centrifuged for 20 min at 3000 × *g*, the supernatant was discarded and the WBC or OBC was estimated as the grams of water or oil per g of dry sample (g/g db).

### 2.10. Leavening and Textural Properties of Doughs Containing Prickly Pear Peel Flour (PPPF)

Doughs were prepared by mixing 100 g of each of the above-mentioned flour blends with 60 mL of SDW, 1.5 g of sodium chloride (NaCl) and 1.5 g of baker’s yeast (*S. cerevisiae*). After kneading, the doughs were transferred to a graduated cylinder and incubated at 30 °C for 90 min, in accordance with the common bakery practice in the manufacturing of bread by the Chorleywood Bread Process (CBP) [25,26]. The leavening behavior of each dough was evaluated after 30, 60 and 90 min of incubation comparing the initial volume to the volume measured after each incubation period [27]. The dough increase percentage (DI%) during leavening time was expressed as the mean value of three repetitions ± standard deviation. The textural parameters of doughs containing different amount of PPPF were evaluated in samples which were let to incubate in a bowl at 30 °C for 90 min, by using the Texture Analyzer Zwick/Roell model Z010 (Zwick Roell Italia S.r.l., Genova, Italy) equipped with an aluminum rectangular probe (5 cm × 4 cm). Each dough was placed between two parallel plates and compressed to 50% of its original height at a speed of 10 mm/s, with a pre-load of 0.01 N and cell load of 50 N, with a two-compression cycle. The samples were evaluated on their hardness (N), cohesiveness (ratio) and springiness (cm), representing, respectively, the peak force of first compression cycle (Fmax), the degree to which the sample can be deformed before its ruptures and the ability of the sample to recover its original form. The results recorded were expressed as the mean ± standard deviation of three replicates obtained using one dough for each measurement.

### 2.11. Determination of the pH and Titratable Acidity of Dough

The pH and titratable acidity of the different doughs, prepared as described before, were evaluated on the unfermented dough (immediately after kneading) and on the leavening of the dough after 30, 60 and 90 min let at 30 °C. The pH variation of each sample was measure by placing a pH probe (Eutech pH 700 Meter) directly in the sample to be analyzed. The total titratable acidity was determined in each sample by homogenizing 10 g of dough with 90 mL of distilled water. After all the dough has dissolved, NaOH 0.1 M was used to titrate 100 g of the sample to pH 8.3.

Each sample was analyzed in triplicate and the results were expressed as the volume (mL) of NaOH required to titrate 100 g of the sample ± standard deviation.

### 2.12. Bread Preparation

The bread was prepared using the bread machine Imetec 7815 Zero Glu (TENACTA, Azzano S. Paolo, BG, Italy). The bread formulations were prepared on the basis of the previous test effectuated on dough. Different samples were prepared by mixing 250 g of durum wheat flour containing 0, 5, 10, 15 and 20% PPPF with 150 mL distilled water, 8.3 g vegetable oil, 3.75 g yeast and 3.75 g salt. All the sample were cooked at the same conditions according to machine program—the baking time was 65 min at 220 °C.

The bread samples were cooled at room temperature (22 °C ± 1), sealed in macro-perforated plastic bags (PA/PE/20/70) (PA: polyamide; PE: polyethylene) (air-packaged) and evaluated the same day.

### 2.13. Physicochemical Properties of Breads

The physical characterization was carried out on the same day as the bakery preparation of bread samples containing 0, 5, 10, 15 and 20% PPPF.

The volume was measured using the rapeseed displacement method, as reported by Spina et al. [28]. The specific volume (cm^3^/g) of each sample, and relative repetition, was calculated as the loaf volume/bread weight.

The moisture content (UM%) was determined on grounded samples by the gravimetric method by drying the sample at 105 °C until they were a constant weight. The Aw was measured, on grounded samples, according to the manufacturer’s instructions, using the hygrometric method at 20 °C by Aqualab Vapor Sorption (Decago Device, Pullman, WA, USA).

### 2.14. Effect of Cooking on Bioactive Compounds

The baked bread samples were cooled at room temperature and their bioactive compounds were evaluated.

Each bread sample containing 0, 5, 10, 15 and 20% PPPF were subjected to an aqueous extraction, as mentioned above, and their total polyphenols, betacyanin, betaxanthin, flavonoid contents were evaluated by using the previously described methods to evaluate the bioactive compounds in PPPF. Each bread sample, containing a different amount of PPPF, was evaluated in triplicate and the results were expressed as the mean value ± standard deviation.

### 2.15. Bread Colour and Texture Evaluation

A color analysis of bread containing different amount of PPPF was conducted by using a portable colorimeter Konica Minolta CM-2500d (Bremen, Germany), using an illuminant D65.

The CIE L*a*b* parameters—the lightness (L*), redness (a*) and yellowness (b*) and psychometric correlates of chroma and hue angle—were determined both on the crust and crumb.

The psychometric correlates of chroma (C) and hue angle (h), were calculated using Equation (1):

(1) $$C={\left.\left({a⚹}^{2}\;+{\;b⚹}^{2}\right.\right)}^{1/2}\;h=\tan^{-1}\left(\frac{b⚹}{a⚹}\right)$$

The color differences among bread samples with different amounts of PPPF were expressed as ΔE, which was calculated using Equation (2):

(2) $$\Delta E=\sqrt[{}]{\left[{\left(L_{x}-L_{0}\right)}^{2}+{\left(a_{x}-a_{0}\right)}^{2}+{\left(b_{x}-b_{0}\right)}^{2}\right]}$$

where subscript “x” indicates the color of the bread formulated with 0, 5, 10, 15 or 20% PPPF and the subscript “0” indicates the color of the control sample.

The textural properties of bread containing different amount of PPPF were analyzed using a Texture Analyzer Zwick/Roell model Z010 (Zwick Roell Italia S.r.l., Genova, Italy) equipped with a cylindrical probe. Bread slices, of 2.5 cm in thickness, were placed between the testing machine and compressed two times to 50% of its original height.

The trial specifications for the textural analysis were a pre-load of 0.01 N, cell load of 50 N, and a cross head speed constant of 10 mm/s. Each sample was placed on a support plate, located inside the testing machine, under the same conditions used for the dough samples.

The results, representing the average of three replicates per sample, were the hardness (N), springiness (cm) and cohesiveness (ratio). Each experiment was repeated twice.

### 2.16. Sensory Analysis

A sensory evaluation was performed using descriptive analysis. The sensory profile of bread samples was determined according to the UNI EN ISO 13299 [29] method and was carried out by 12 trained panelists with several years of tasting experience and who have been frequently used in our previous studies on breads. The panelists choose to participate in the research and signed the informed consent as our institution does not have an ethics committee for taste and food quality evaluation studies.

The judges selected a list of descriptors for the sensory profile using handmade breads [2,28]. A detailed definition (Table 1) was established for each sensory attribute [30]. The selected attributes described the texture and flavor characteristics as extensively as possible. The judges evaluated the intensity of the selected sensory attributes using a scale between 1 (absence of the sensation) and 9 (extremely intense) (Table 1) (FIZZ Byosistemes, ver.2.00 M, Couternon, France). The data reported were expressed as the mean ± standard deviation.

The bread samples containing 0, 5, 10, 15 and 20% PPPF were evaluated in the equipped laboratory [31] of the Dipartimento di Agricoltura Alimentazione e Ambiente (Di3A) in individual booths illuminated with white light served at an ambient temperature (22 ± 2 °C) and identified with a random three-digit code. The breads were sliced (slices 15 mm thick) ten minutes before tasting. The first and the last slices of the loaves were discarded. The judges, between sample evaluations, rinsed their mouth with water.

### 2.17. Statistical Analysis

Data, expressed as the mean ± standard deviation, were statistically analyzed by using the statistical package software Minitab™ version 16.0. The significant effect of different PPPF amounts was determined with a one-way ANOVA (*p* < 0.05) and significant (*p* < 0.05) differences (mean separation) between samples were determined by Fisher’s least significant difference (LSD) test.

## 3. Results and Discussion

### 3.1. Nutritional Composition and Bioactive Compounds of Prickly Pear Peel Flour (PPPF)

Table 2 shows the composition of PPPF in terms of its nutritional and target bioactive compounds, obtained as described before. In detail, PPPF evidenced a low moisture percentage (M%) value and a low water activity (Aw), registering 8.17 ± 0.05% and 0.34 ± 0.00, respectively.

The flour exhibited a good protein and fat content of 3.58 ± 0.03% and 2.12 ± 0.02%, respectively. Moreover, the prickly pear peel evidenced an elevated amount of total dietary fiber, reaching the value of 33.00 ± 00%, in accordance with Anwar and Sallam [14]. Moreover, considering a total fiber content of 33% for PPPF, the addition of 15% and 20% PPPF obtains a total fiber content of 8.01% and 9.48%, respectively, in the blend comparable to whole wheat flour (8.4%).

The analysis of bioactive compounds reveals that PPPF contains a high amount of polyphenols (17.1 ± 0.9 mg GAE/g). A very similar content was reported also by Mahloko et al. [32], who suggest that a high TPC is related to ascorbic acid and other antioxidant contents, such as pectin, carotenes, betalains, quercetin and their derivatives.

Moreover, the spectrophotometric analysis of betaxanthins and betacyanins shows that PPPF has a high concentration of yellow pigments compared to that of red pigments.

To our knowledge, few authors have investigated the content of phenolic compounds and betalains in prickly pear peels. Melgar et al. [33] reported a characterization of the betalain in different species of *Opuntia ficus indica* var. gialla e sanguigna by LC-DAD-ESI/MSn and Gomez-Maqueo et al. The study of [34] describes the release mechanism of bioactive compounds under a high hydrostatic pressure.

### 3.2. Evaluation of Doughs Containing Prickly Pear Peel Flours

#### 3.2.1. Water and Oil Absorption Capacity

**Figure 1:**
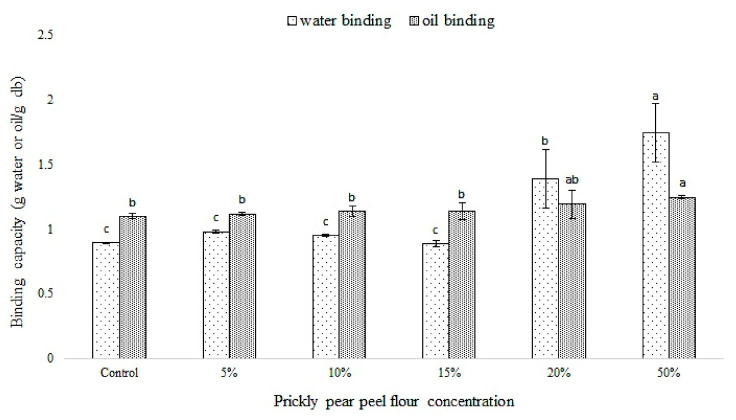
Water and oil absorption capacity of different blend performed with durum wheat flour and 5, 10, 15, 20 or 50% of prickly pear peel flour (PPPF). Control was made with durum wheat flour, without any PPPF addition. Within the same parameters (water binding or oil binding), columns followed by different letters are significantly different according to Fisher’s least significant difference test (*p* < 0.05). Vertical bars indicate the standard deviation of the mean.

Figure 1 displays the water and oil absorption capacity of composite flours performed with durum wheat flour mixed with 5, 10, 15, 20 and 50% PPPF.

Regarding the WBC, no significant differences (*p* > 0.05) were observed between the control sample performed with only durum wheat (0%) and those containing 5, 10 and 15% PPPF. The increasing water absorption capacity was registered only in the blend containing 20 and 50% PPPF and, among them, this latest showed the (*p* < 0.05) highest significant value (Figure 1). Similar results were obtained when the samples were evaluated on their capacity to absorb the oil. The highest significant oil absorption capacity (*p* < 0.05) was registered in the sample containing 50% PPPF, followed by the sample containing 20% PPPF. No significant differences (*p* > 0.05) were observed among the other samples (Figure 1). The results obtained are in accordance with many authors that reported that the fiber content belonging to a vegetable matrix led to increase the water holding capacity, changing the dough properties [32].

#### 3.2.2. Leavening Capacity and Textural Properties

**Figure 2:**
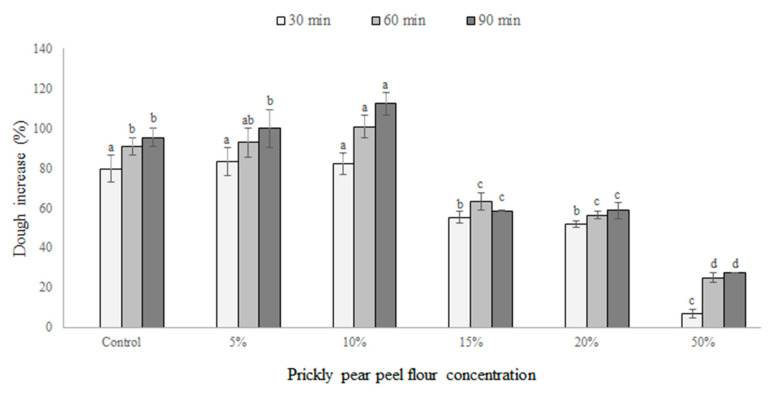
Leavening capacity of doughs added with different prickly pear flour concentrations and evaluated after 30, 60 and 90 min of incubation at 30 ± 1 °C. Columns at the same incubation time (30, 60 or 90 min) followed by different letters are significantly different according to Fisher’s least significant difference test (*p* < 0.05). Vertical bars indicate the standard deviation of the mean.

Figure 2 displayed the dough increase percentage (DI%) of samples performed with different amounts of PPPF and evaluated after 30, 60 and 90 min of incubation at 30 °C. After 30 min of incubation, both the control (0%) and samples with 5 and 10% PPPF evidenced a significant (*p* < 0.05) highest DI% value. At the same time point, the dough made with 15 and 20% PPPF evidenced a lower, but still acceptable, DI% value [35], while the sample with 50% PPPF showed the lowest DI% increase.

After 60 and 90 min of incubation, the sample containing 10% PPPF registered the significant (*p* < 0.05) highest DI% value of, even surpassing the control (0%). After 90 min, although the values were lower than the control, the dough with 15 and 20% PPPF exhibited a higher dough increase percentage. The inclusion of 50% PPPF significantly (*p* < 0.05) reduced the DI% recording non-appreciable values even after 90 min of incubation (Figure 2).

The 10% prickly pear flour concentration probably provides the optimal nutrient sources for the growth and CO_2_ production of *S. cerevisiae* [36,37,38].

Aboaba and Obakpolor [39] reported a dough-containing concentration up to 20% of cassava flour significantly reduced the volume of dough during leavening, probably due to the fact that as the concentration of wheat flour in each successive sample was reduced, the concentration of wheat gluten was also reduced and thus a corresponding decrease was seen in the dough volume.

Thus, a reduction in gluten, responsible for the entrapment of carbon dioxide, progressively caused a reduction in the DI% in our sample.

The textural analysis (hardness, cohesiveness and springiness) of dough performed with different amounts of PPPF and let to incubate at 30 °C for 90 min is reported in Table 3. The addition of PPPF significantly influenced (*p* < 0.05) the hardness of the sample when it reaches high concentrations. In particular, non-significant (*p* > 0.05) differences were observed between the control (0%) and samples containing 5, 10 and 15% PPPF while the hardness increased significantly (*p* < 0.05) in the sample containing 20% PPPF, reaching the highest value in the sample containing 50% PPPF (Table 3). The increasing hardness in the samples containing 20 and 50% PPPF can be correlated with the previously reported WBC (Figure 1) of each blend. In fact, as reported Wu et al. [40], the hardness increases when dough shows a relatively low water distribution, probably due to the ability of fiber to absorb water. Among samples, the dough made with 20% PPPF showed the highest significant (*p* < 0.05) cohesiveness value, whereas the highest significant (*p* < 0.05) springiness value was recorded in the sample containing 50% PPPF.

Our results are in accordance with those reported by Ayadi et al. [41], who evidenced that the cohesion values of dough containing cladode powders are only minimally influenced at the highest concentration of 20%, while at the same concentration the hardness of dough significantly increases.

#### 3.2.3. pH and Titratable Acidity Determination

The addition of PPPF in bread formulation caused a significant (*p* < 0.05) decrease in the dough pH, during all incubation times considered (after 0, 30, 60 and 90 min) (Table 4). In particular, after dough preparation (time 0), with an increasing PPPF concentration up to 50%, the dough pH decreased from 5.57 ± 0.03 (control) to 5.13 ± 0.02. After 90 min of incubation, the pH decreased from 5.59 ± 0.03 (control) to 5.03 ± 0.03 (50% PPPF).

The monitored titratable acidity (Table 4) values evidenced a significant (*p* < 0.05) increment with an increase in the PPPF concentration, probably due to the high acid ascorbic acid generally presented in this matrix [42]. Samples containing 15, 20 and 50% PPPF evidenced the highest values, even at time zero, followed by the sample containing 10%. The lowest values were detected in sample containing 5% PPPF and in the control during all incubation times.

### 3.3. Evaluation of Bread Containing Prickly Pear Peel Flours

#### 3.3.1. Physicochemical Characterization

Table 5 reports the physical properties of the breads containing the same amount of PPPF previously used for dough formulation. Only PPPF at 50% was not used for bread preparation because the previous assay revealed a non-appreciable increase in the leavening dough capacity even after 90 min of incubation.

Regarding the weight, no significant differences (*p* > 0.05) were observed among the bread samples, independently from the level of flour added. The bread containing 10% PPPF showed the highest significant (*p* < 0.05) volume (cm^3^) and specific volume (cm^3^/g), followed by the control and sample containing 5% PPPF, whereas the lowest values were measured in the samples containing 15 and 20% PPPF. Contrariwise, the 15 and 20% PPPF samples evidenced the highest significant (*p* < 0.05) values of M% followed by the control. The sample containing 10% PPPF was found to have the lowest values of M% and Aw, which is a desirable feature in order to reduce the microorganism spoilage and to increase the potential shelf-life [32].

#### 3.3.2. Texture

**Figure 3:**
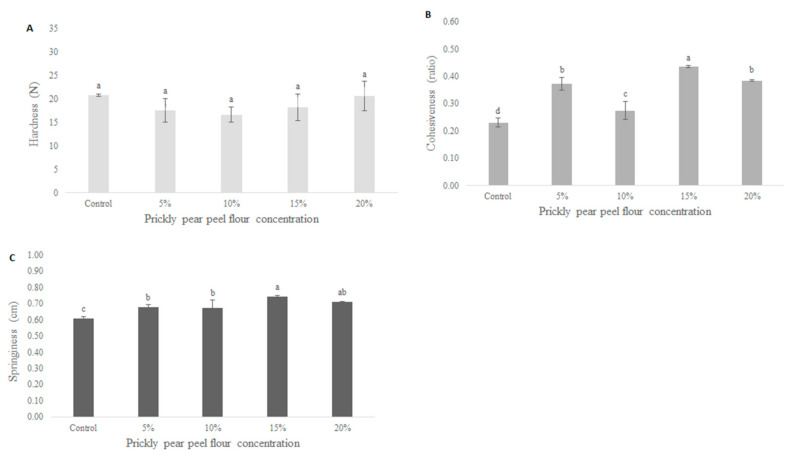
Effect of prickly pear peel flour concentration on the bread parameters: hardness (**A**), cohesiveness (**B**) and springiness (**C**). Columns followed by different letters are significantly different according to Fisher’s least significant difference test (*p* < 0.05). Vertical bars indicate the standard deviation of the mean.

Figure 3 displays the textural parameters of bread performed with different percentages of PPPF. The inclusion of PPPF involved a significant change in hardness, although the bread with 10% PPPF registered the lowest value which is probably directly related to the highest values of specific volume previously reported. As reported by many authors [43,44], a more compact crumb is strictly related to a small bread volume, which frequently determines higher hardness values. The cohesiveness registered the lowest significant (*p* < 0.05) value in the control sample followed by the sample with 10% PPPF with the values of 0.23 ± 0.02 and 0.27 ± 0.03, respectively, indicating a low resistance of these samples to resist at deformation before the point of break. The springiness of the bread registered a lowest significant (*p* < 0.05) value in the control sample (0.61 ± 0.01), followed by the bread samples containing 5 and 10% PPPF (0.68 ± 0.02 and 0.67 ± 0.05, respectively). High springiness values are attributed to the interactions between gelatinized starch and gluten dough that, due to the high temperature, can form a sponge structure that makes the sample more elastic [45]. The high springiness and the deriving reduction in elasticity is more evident in samples containing 15 and 20% PPPF, probably due to a reduction in the gluten content since prickly pear peel does not contain it.

#### 3.3.3. Color

Table 6 shows the color parameter values of bread containing several PPPF additions, evaluated on both crust and crumb sections.

The crust of samples supplemented with PPPF, in comparison to the control, attested a decrease in the lightness (L*) and yellowness (b*) parameters, especially in samples contain 15 and 20% PPPF, which showed lowest significant (*p* < 0.05) values in comparison to the control. Otherwise, the samples containing 10, 15 and 20% PPPF, highlighted a significant (*p* < 0.05) increase in the crust redness (a*). The crust color evaluation shows the highest significant (*p* < 0.05) C and hue angle (h) values, measuring, respectively, the color saturation and relative amounts of redness and yellowness in samples containing 15 and 20% PPPF, in comparison to the control.

The L* parameter, evaluated on crumb, significantly (*p* < 0.05) decreased in samples containing PPPF concentrations above 5%, while all increasing additions of PPFF (5, 10, 15 and 20%) resulted in a progressive significant (*p* < 0.05) increase in a*, b* and C parameters. The h value was also significantly (*p* < 0.05) influenced by even the smallest percentage of PPPF which determined a decrease in the values.

Sapers and Hornstein [46] reported that the b* value is strictly correlated to betaxanthin–betacyanin content. Our data show how, in the crust, the b* value decreases, probably due to the thermolability of these pigments [47] which is most exposed to the high temperature. In the crumb, the L* value increases progressively as the PPPF concentration increases, suggesting a protective effect of the bread matrix towards these pigments (Table 6).

Moreover, the ΔE values indicated that the differences between the control and samples with added PPPF where “very distinct” both in the crust and crumb, assuming values are >3 even when the control was compared with bread samples containing the lowest PPPF concentration (5%) (data not shown).

As reported by Francis and Clydesdale [48] samples can be considered “without perceptible differences”, “distinct” or “very distinct” when ΔE < 1.5, 1.5 < ΔE < 3 and ΔE > 3, respectively.

#### 3.3.4. Recovery of Bioactive Compounds

The results regarding the bioactive compounds of bread samples supplemented with PPPF are given in Table 7. The addition of PPPF significantly (*p* < 0.05) affects the total polyphenols and flavonoid content of the bread containing 10, 15 and 20% PPPF while no significant (*p* > 0.05) differences were observed between the control and samples containing 5% PPPF. The highest significant (*p* < 0.05) value in betalanin and betaxanthin compounds was observed in the sample containing 20% PPPF, followed by the samples containing 15, 10 and 5%. Moreover, even the lowest concentration of PPPF (5%) led to a significant (*p* < 0.05) increase in the antioxidant activity of the sample. A similar trend was obtained by Elhassaneen et al. [49], whereby the addition of prickly pear peel flour at 5%, for biscuit preparation, improved the TPC content with respect to the control after cooking. The same was observed by Mahloko et al. [32] with prickly pear and banana peel flours. In our study, good results were obtained regarding the stability of the bioactive molecules. By comparing the bioactive compounds and antioxidant activity determined in PPPF (Table 2) with those obtained in bread samples containing different amounts of PPPF (Table 7), it is evident that the thermal process led to an increase in the betalain content and antioxidant activity, probably due to a better extraction which occurs in the bread matrix during cooking. Additionally, Slavov et al. [50] reported that a sample of red beet juice, subjected to microwave treatment, showed the highest total betalains content and a significant increase in antioxidant activity.

#### 3.3.5. Sensory Evaluation

A total of 24 sensory attributes were selected by the panelists for the descriptive analysis of bread samples. They included two attributes for appearance, five for both odor and flavor, five for taste, six for texture and an overall judgment. Of the 24 attributes, 18 significantly differentiate two or more of the samples (Table 8).

The two appearance attributes: crumb color and alveolation uniformity differentiate the control bread from one or more bread samples.

Increasing the levels of PPPF significantly differentiated the crumb color of the control sample from the other samples. The crumb color rating changes from 2.00 in the control bread to 7.64 in bread with 20% PPPF. The crumb color is highly related to the ingredients and recipe [30]. The addition of PPPF increased the color intensity of the crumbs, in agreement with the trend of the reported color parameters (see Section 3.3.3).

The alveolation was fine, homogeneous in bread samples with PPPF, and similar to that of the control bread with the exception of bread with 10% PPPF, suggesting a weak effect of the PPPF at its highest concentration. The characteristic aroma of bread is certainly one of the most important parameters influencing its acceptance by consumers [51]. The addition of PPPF up to 10% did not differentiate the fortified bread samples from control bread. Bread with 15 and 20% of PPPF received a significantly lower rating of bread odor than the control. In addition, the control bread and bread with PPPF up to 10% were similar for fruity and green/grassy odor attributes. Fortification at the highest levels of PPPF induces a significant increase in the rating of both attributes. The intensity of the odor of yeasty was perceived similar between control bread and the other bread samples.

As well as for the bread odor attribute, the most perceived flavor in all samples was the flavor of bread. The other flavor attributes were perceived with lower intensities. The highest levels of PPPF in bread (15–20%) differentiate these samples from the control bread for the bread and fruity flavor attributes. The increased intensity of the fruity flavor probably reduced/covered the perceived flavor of bread.

Bread samples fortified with PPPF (10–20%) were perceived differently with respect to the control and bread with 5% PPPF, in terms of the characteristic green/grassy flavor, due to the contribution of the PPPF.

Notwithstanding the effect of the addition of PPPF on flavor attributes, the bread with PPPF was similar to the control bread both in terms of yeasty and off flavors.

As concern taste attributes, sweet and salty flavors were those perceived with the highest intensity, but the salty flavor was similar in all samples. The addition of PPPF increased the sweet rating and the sample with 20% PPPF was different from the control. Sour, bitter and astringency flavors were generally perceived as weak by the panelists. A different rating differentiates the control bread from samples with 15% PPPF for a sour flavor and from samples with 20% PPPF for bitter and astringency flavors.

The addition of PPPF seems not to influence the textural attributes. Concerning surface moistness, only bread with 5% PPPF was different from control bread, and bread with 15% PPPF was different for cohesiveness, respect to the control bread. The presence of PPPF in bread did not make a difference in the control bread’s cohesiveness in comparison to the other samples. No significant differences were found between the control bread and the other bread samples for the texture attributes, such as the dryness, coarse/grittiness, and chewiness. Moreover, the overall evaluation of bread samples was similar.

These results suggest that the addition of PPPF produces a direct effect on the crumb color, while for the other sensory attributes, when affected, they were influenced only by the addition of 15–20% of PPPF.

## 4. Conclusions

The prickly pear peel, which is considered to be a by-product, can obtain vegetable flour rich in bioactive compounds and with a high antioxidant activity. The PPPF also was revealed to be a good source of dietary fiber that improves the nutritional characteristics of bread, allowing for a functional product. Furthermore, the recovery of bioactive compounds also revealed a high amount of total polyphenols and betalains after the baking process. Among the different formulations, the replacement of the wheat flour with 10% PPPF leads to obtain the highest dough increase and the best results in terms of the specific volume. Moreover, the sensory characteristics of bread formulated with the 10% PPPF registered the highest total sensory evaluation scores.

## Figures and Tables

**Table 1:** Sensory attributes, definition and anchors used in the descriptive analysis of bread samples.

Attribute	Definition	Scale Anchors	
		1	9
*Visual appearance*			
Crumb Color	Strength of color from light to dark	Light	Dark
Alveolation uniformity (crumb)	Porosity and homogeneity of the size of the holes	Fine and very homogeneous	Heterogeneous
*Odor/flavor attributes*			
Bread	Intensity of the characteristic odor/flavor of freshly baked bread	weak	strong
Yeasty (crumb)	Intensity of the characteristic odor/flavor associated to yeast used as leaving agent	weak	strong
Fruity	Intensity of the characteristic odor/flavor associated with general fruit	weak	strong
Green/Grassy	Intensity of the characteristic odor/flavor of unripe fruit, green grass or weeds.	weak	strong
Off odour/Off flavour	Odor/flavor unpleasant, not characteristic of bread	weak	strong
*Taste attributes*			
Sweet	Primary sensation produced by sugars	weak	strong
Salty	Primary sensation produced by sodium chloride	weak	strong
Sour	Primary sensation produced by citric acid	weak	strong
Bitter	Primary sensation produced by caffeine.	weak	strong
Astringency	Sensations of shrinking, puckering or roughing in the mouth	weak	strong
*Texture attributes*			
Surface moistness	Degree of moistness perceived on the surface of the product when in contact with the lips	Dry	Wet
Softness	Degree of softness in mouth	Soft	Hard
Cohesiveness	Degree to which the chewed sample holds together	weak	strong (cohesive mass)
Dryness	Degree of drying effect, amount of saliva absorbed by the sample during chewing	weak	strong
Coarse/Grittiness	Degree of particle perception on tongue Degree of the presence of small insoluble particles in the mouth after ingesting the sample	weak	strong
Chewiness	Number of chews required before swallowing	few	several
Overall	Degree of the overall assessment considering all of the attributes	low	high

**Table 2:** Nutritional and functional characterization of prickly pear peel flour (PPPF).

Nutritional Composition		Bioactive Compounds ^1^	
Parameters	PPPF	Parameters	PPPF
Moisture (%)	8.17 ± 0.05	Total polyphenols (mg/g)	17.1 ± 0.9
Water activity (Aw)	0.34 ± 0.00	Flavonoid (mg/100 g)	16.9 ± 2.7
Ash (%)	12.71 ± 0.03	Betacyanins (mg/100 g)	7.60 ± 0.7
Protein (%)	3.58 ± 0.03	Betaxanthin (mg/100 g)	12.0 ± 1.3
Fat (%)	2.12 ± 0.02	DPPH (mg/kg)	6.44 ± 0.02
Total carbohydrates (%)	73.41 ± 0.02		
Reducing sugars (%)	43.58 ± 2.62		
Total dietary fiber (%)	33.00 ± 0.0		
Soluble dietary fiber (%)	12.80 ± 0.2		

^1^ Values expressed on dry matter.

**Table 3:** Textural properties of dough incorporating prickly pear peel flour (PPPF).

PPPF %	Textural Parameters		
	Hardness (N)	Cohesiveness	Springiness (cm)
Control	1.60 ± 0.23c	0.29 ± 0.01b	0.30 ± 0.01ab
5	1.63 ± 0.16c	0.27 ± 0.04b	0.28 ± 0.05b
10	1.79 ± 0.27c	0.30 ± 0.01b	0.29 ± 0.00b
15	1.92 ± 0.14c	0.31 ± 0.01ab	0.32 ± 0.01ab
20	2.35 ± 0.01b	0.35 ± 0.06a	0.29 ± 0.00ab
50	2.81 ± 0.80a	0.30 ± 0.11b	0.33 ± 0.11a

Data expressed as the mean ± standard deviation. In each column, values followed by different letters are significantly different (*p* < 0.05).

**Table 4:** pH and titratable acidity of dough samples content in bread samples at different times of storage.

PPPF %	Time 0		Time 30		Time 60		Time 90	
	pH	Acidity %	pH	Acidity %	pH	Acidity %	pH	Acidity %
Control	5.57 ± 0.03a	5.40 ± 1.00c	5.65 ± 0.02a	4.10 ± 0.50d	5.69 ± 0.02a	3.65 ± 0.25f	5.59 ± 0.03a	2.65 ± 0.35e
5	5.48 ± 0.05b	4.90 ± 1.60c	5.53 ± 0.04b	5.60 ± 0.10cd	5.47 ± 0.04b	6.65 ± 0.05e	5.50 ± 0.06a	3.50 ± 0.20e
10	5.57 ± 0.01a	9.23 ± 0.59b	5.48 ± 0.07b	7.45 ± 2.35c	5.36 ± 0.03c	8.15 ± 0.05d	5.38 ± 0.10b	6.20 ± 0.40d
15	5.52 ± 0.04ab	11.75 ± 1.25a	5.29 ± 0.02c	10.70 ± 0.50b	5.18 ± 0.02e	10.30 ± 0.10c	5.14 ± 0.03d	7.25 ± 1.15c
20	5.34 ± 0.02c	13.15 ± 0.65a	5.28 ± 0.04c	10.20 ± 1.30b	5.26 ± 0.02d	15.45 ± 0.15b	5.24 ± 0.01c	8.90 ± 0.10b
50	5.13 ± 0.02d	13.40 ± 1.30a	5.06 ± 0.02d	12.90 ± 0.00a	5.04 ± 0.04f	16.90 ± 0.20a	5.03 ± 0.03e	17.25 ± 0.25a

Data expressed as the mean ± standard deviation. In each column, values followed by different letters are significantly different (*p* < 0.05).

**Table 5:** Properties of bread samples containing different percentages of PPPF.

	Parameters				
PPPF%	Weight (g)	Volume (cm^3^)	Specific volume (cm^3^/g)	M%	Aw
Control	52.06 ± 0.76a	136.9 ± 20.1b	2.63 ± 0.39b	24.09 ± 0.50b	0.91 ± 0.01a
5%	52.49 ± 0.69a	132.6 ± 22.9b	2.52 ± 0.42b	22.99 ± 0.71c	0.89 ± 0.01b
10%	52.01 ± 1.04a	160.6 ± 11.9a	3.09 ± 0.24a	20.90 ± 0.70d	0.88 ± 0.01c
15%	52.71 ± 1.21a	102.8 ± 19.5c	1.95 ± 0.38c	26.91 ± 0.46a	0.90 ± 0.00ab
20%	51.70 ± 0.57a	118.8 ± 15.0bc	2.30 ± 0.29bc	26.93 ± 0.64a	0.88 ± 0.00c

Data expressed as the mean ± standard deviation. In each column, values followed by different letters are significantly different (*p* < 0.05).

**Table 6:** Color parameters in bread samples for crust and crumb.

	Color Parameters									
	Crust					Crumb				
PPPF%	L*	a*	b*	C	h	L*	a*	b*	C	h
Control	66.6 ± 1.3a	6.5 ± 0.93c	34.4 ± 1.9ab	35.0 ± 2.0ab	79.3 ± 1.3a	65.5 ± 3.0a	−2.9 ± 0.3e	21.9 ± 1.0e	22.1 ± 1.0e	97.6 ± 0.8a
5%	60.1 ± 5.0b	8.4 ± 2.9b	35.6 ± 2.3a	36.6 ±1.9a	76.7 ± 5.0a	66.6 ± 1.7a	−2.7 ± 0.2d	25.0 ± 0.7d	25.1 ± 0.7d	96.1 ± 0.5b
10%	52.5 ± 5.1c	12.0 ± 1.4a	32.0 ± 4.3bc	34.3 ± 3.9ab	69.1 ± 3.8b	62.3 ± 3.1b	−1.5 ± 0.4c	29.1 ± 1.4c	29.1 ± 1.4c	92.9 ± 0.9c
15%	51.7 ± 5.8cd	11.6 ± 1.9a	31.4 ± 3.2c	33.6 ± 2.5b	69.5 ± 4.6b	61.6 ± 1.4b	−0.5 ± 0.1b	31.8 ± 0.7b	31.8 ± 0.7b	90.1 ± 0.3d
20%	47.9 ± 5.4d	11.8 ± 1.7a	30.5 ± 5.2c	32.8 ± 4.5b	68.3 ± 5.3b	57.9 ± 1.5c	0.93 ± 0.3a	33.1 ± 0.8a	33.2 ± 0.8a	88.4 ± 0.5e

Data expressed as the mean ± standard deviation. In each column, values followed by different letters are significantly different (*p* < 0.05).

**Table 7:** Bioactive compound of bread containing different amounts of PPPF.

Parameters	Bread Containing Different Percentage (%) of PPPF				
	Control	5%	10%	15%	20%
Total polyphenols (mg/g)	0.44 ± 0.15d	0.55 ± 0.23d	1.44 ± 0.21c	2.59 ± 0.46b	3.98 ± 0.21a
Flavonoid (mg/100g)	2.87 ± 0.42c	3.07 ± 0.42c	4.67 ± 0.88b	6.36 ± 1.39a	7.34 ± 1.35a
Betacyanins (mg/kg)	0.00 ± 0.00e	8.33 ± 2.82d	24.16 ± 1.40c	28.06 ± 0.69b	32.10 ± 2.05a
Betaxanthin (mg/kg)	0.00 ± 0.00e	7.43 ± 1.67d	22.30 ± 3.38c	29.98 ± 0.83b	36.27 ± 1.38a
DPPH (mg/kg)	0.00 ± 0.00d	0.23 ± 0.05cd	0.47 ± 0.02bc	0.67 ± 0.43b	1.62 ± 0.03a

Data expressed as the mean ± standard deviation. In each row, values followed by different letter within the same parameter are significantly different according to the Fisher’s least significant difference test (*p* ≤ 0.05).

**Table 8:** Sensory profile of bread samples.

Attribute	Bread Samples				
	Control	5%PPPF	10%PPPF	15%PPPF	20%PPPF
*Visual appearance*					
Crumb color	2.00 ± 0.89d	3.36 ± 1.29c	4.73 ± 1.68b	5.64 ±1.57b	7.64 ±0.81a
Alveolation uniformity	3.73 ± 1.74b	5.09 ± 1.70ab	5.36 ± 1.29a	3.82 ±2.04ab	4.36 ±2.38ab
*Odor attributes*					
Bread	5.55 ± 1.81a	4.55 ± 1.44abc	4.91 ± 2.02ab	3.73 ±2.20bc	3.09 ±2.39c
Yeasty	2.36 ±1.29ab	3.64 ± 2.62a	2.82 ± 1.94ab	2.00 ±1.41b	2.09 ±1.58ab
Fruity	1.18 ± 0.60c	1.82 ± 1.17bc	2.27 ± 1.10abc	2.73 ±2.20ab	3.46 ±2.25a
Green/Grassy	1.27 ± 0.91c	2.00 ± 1.41bc	2.46 ± 1.64abc	2.91 ±1.70ab	3.73 ±2.05a
Off odor	1.36 ± 0.92a	2.09 ± 1.76a	1.64 ± 1.21a	1.73 ±1.27a	2.36 ±2.42a
*Flavor attributes*					
Bread	4.64 ± 2.16a	4.82 ± 0.75a	4.46 ± 1.44a	3.55 ±1.51ab	2.91 ±1.97b
Yeasty	1.82 ± 1.08ab	2.09 ± 1.14ab	2.18 ± 1.17a	1.82 ±1.25ab	1.27 ±0.47b
Fruity	1.18 ± 0.60b	2.64 ± 2.06ab	2.82 ± 2.32ab	3.09 ±2.26a	4.09 ±2.55a
Green/Grassy	1.27 ± 0.91c	1.55 ± 0.82bc	3.18 ± 2.40a	2.64 ±1.91abc	2.91 ±1.76ab
Off flavor	1.46 ± 1.04ab	1.82 ± 1.40ab	1.36 ± 0.92b	2.46 ±1.64a	1.73 ±1.01ab
*Taste attributes*					
Sweet	2.36 ± 1.36b	1.82 ± 1.08b	1.73 ± 1.01b	3.00 ± 1.90ab	3.91 ±2.21a
Salty	2.73 ± 1.90	3.00 ± 1.18	3.09 ± 1.04	2.36 ±1.21	2.00 ±1.10
Sour	1.09 ± 0.30b	1.46 ± 0.69ab	1.73 ± 1.19ab	2.09 ±1.22a	2.00 ±1.00a
Bitter	1.64 ± 1.43b	2.09 ± 2.07b	2.09 ± 1.30b	2.82 ±1.33ab	3.64 ±2.11a
Astringency	1.64 ± 1.03b	1.55 ± 0.82b	2.18 ± 1.47ab	1.82 ±0.87ab	3.00 ±2.57a
*Texture attributes*					
Surface moistness	3.09 ± 1.04b	4.55 ± 1.29a	3.82 ± 1.83ab	4.18 ±1.54ab	4.27 ±2.28ab
Softness	3.64 ± 2.20ab	5.00 ± 2.37a	3.82 ± 1.78ab	4.36 ±1.96ab	3.27 ±1.56b
Cohesiveness	3.09 ± 2.12b	4.27 ± 1.74ab	4.36 ± 1.63ab	5.00 ±1.79a	3.73 ±0.91ab
Dryness	3.73 ± 2.20a	2.91 ± 1.87a	3.18 ± 1.40a	2.55 ±1.37a	3.09 ±1.30a
Coarse/Grittiness	1.73 ± 0.79a	1.82 ± 1.25a	1.91 ± 1.22a	1.64 ±0.92a	2.36 ±2.16a
Chewiness	4.64 ± 2.11a	4.27 ± 1.85a	4.09 ± 1.30a	4.82 ±1.94a	4.73 ±1.79a
Overall	5.82 ± 1.25a	6.27 ± 1.74a	6.55 ± 1.29a	5.91 ±2.07a	5.56 ±2.42a

Data expressed as the mean ± standard deviation. In each row, values followed by different letter within the same parameter are significantly different according to the Fisher’s least significant difference test (*p* ≤ 0.05).
